# Functional *FGFR4* Gly388Arg polymorphism contributes to cancer susceptibility: Evidence from meta-analysis

**DOI:** 10.18632/oncotarget.15811

**Published:** 2017-02-28

**Authors:** Si-Wei Xiong, Jianqun Ma, Fen Feng, Wen Fu, Shan-Rong Shu, Tianjiao Ma, Caixia Wu, Guo-Chang Liu, Jinhong Zhu

**Affiliations:** ^1^ Department of Urology, Guangzhou First People's Hospital, Guangzhou Medical University, Guangzhou 510180, Guangdong, China; ^2^ Department of Thoracic Surgery, Harbin Medical University Cancer Hospital, Harbin 150040, Heilongjiang, China; ^3^ Department of Gastroenterology, The First People's Hospital of Foshan (Affiliated Foshan Hospital of Sun Yat-sen University), Foshan 528000, Guangdong, China; ^4^ Department of Pediatric Urology, Department of Pediatric Surgery, Guangzhou Institute of Pediatrics, Guangzhou Women and Children's Medical Center, Guangzhou Medical University, Guangzhou 510623, Guangdong, China; ^5^ Department of Gynecology and Obstetrics, The First Affiliated Hospital of Jinan University, Guangzhou 510630, Guangdong, China; ^6^ Department of Internal Medicine, Harbin Medical University, Harbin 150081, Heilongjiang, China; ^7^ Molecular Epidemiology Laboratory and Department of Laboratory Medicine, Harbin Medical University Cancer Hospital, Harbin 150040, Heilongjiang, China

**Keywords:** FGFR4, Gly388Arg, polymorphism, cancer, meta-analysis

## Abstract

Fibroblast growth factor receptor 4 (FGFR4) is a member of receptor tyrosine kinase family. A functional Gly388Arg (rs351855 G>A) polymorphism in *FGFR4* gene causes a glycine-to-arginine change at codon 388 within the transmembrane domain of the receptor. Although the *FGFR4* rs351855 G>A polymorphism has been implicated in cancer development, its association with cancer risk remains controversial. Here, we have systematically analyzed the association between the rs351855 G>A polymorphism and cancer risk by performing a meta-analysis of 27 studies consisting of 8,682 cases and 9,731 controls. Odds ratios (ORs) and 95% confidence intervals (CIs) were calculated to measure the strength of the association. The rs351855 G>A polymorphism was associated with an increased cancer risk under the recessive model (OR=1.19, 95% CI=1.01-1.41). Stratified analysis by cancer type indicated the rs351855 G>A polymorphism was associated with an increased risk of breast and prostate cancer, but a decreased risk of lung cancer. This meta-analysis demonstrates the *FGFR* rs351855 G>A polymorphism is associated with increased cancer risk and suggests it could potentially serve as a chemotherapeutic target or biomarker to screen high-risk individuals.

## INTRODUCTION

Cancer represents an enormous economic burden on society in both developing and developed countries. Based on the GLOBOCAN 2012 estimates, there were about 14.1 million new cancer cases in 2012, and 8.2 million deaths [1]. Cancer is a complex multifaceted disease that results from gene-environment interactions. Apart from the lifestyles associated with an increased risk of a number of common cancers, genetic variations, including single nucleotide polymorphism (SNP), have been known to affect cancer susceptibility.

Fibroblast growth factor receptors (FGFRs), composed of four related proteins (FGFR1-4), belong to the receptor tyrosine kinase (RTK) family. To date, more than 18 FGF ligands have been identified [2]. The binding of ligands to FGFRs triggers several downstream signal transduction cascades that are activated in cancer, including phospholipase C (PLC), phosphatidylinositol 3-kinase (PI3K), signal transducer and activator of transcription (STAT), as well as mitogen-activated protein kinases (MAPKs) [3, 4]. FGFRs participate in the regulation of multiple crucial biological activities, including cell proliferation and differentiation, migration, angiogenesis, and survival [3, 5, 6]. Numerous studies have indicated an aberrant FGFR signaling in carcinogenesis [2, 7, 8]. Recently, a large scale analysis of 4,853 solid tumors has revealed that 7.1% of cancers harbor FGFR aberrations, including gene amplifications (66%), mutations (26%), and rearrangements (8%) [9]. Over the past decade, different types of FGFR inhibitors have been developed to treat cancer, including multi-target tyrosine kinase inhibitors, FGFR specific tyrosine kinase inhibitors (TKIs), monoclonal antibodies, and FGF ligand traps [2, 10].

FGFR4 is a highly versatile protein that has more than 20 known ligands [5], and is highly expressed in various types of cancer [11–16]. Overexpression of FGFR4, but not other FGFRs, stimulates membrane ruffling, resulting in increased motility of COS-7 cells [17]. The *FGFR4* gene is highly polymorphic. A common nonsynonymous SNP rs351855, which causes a substitution of arginine instead of glycine in the transmembrane domain of the EGFR4 receptor (Gly388Arg) has been implicated in cancer development [18]. *FGFR4* gene rs351855 G>A polymorphism has been associated with genetic predisposition to several types of cancer, including breast cancer [18–22], prostate cancer [23–26], head and neck cancer [27], lung cancer [28, 29], and hepatocellular carcinoma [13, 30, 31]. Unfortunately, the association between the *FGFR4* gene rs351855 G>A polymorphism and cancer risk remains controversial. To systematically analyze the association between the *FGFR4* gene rs351855 G>A polymorphism and cancer risk, we have performed this meta-analysis using 27 studies consisting of 8,682 cases and 9,731 controls.

## RESULTS

### Literature search

**Figure 1:**
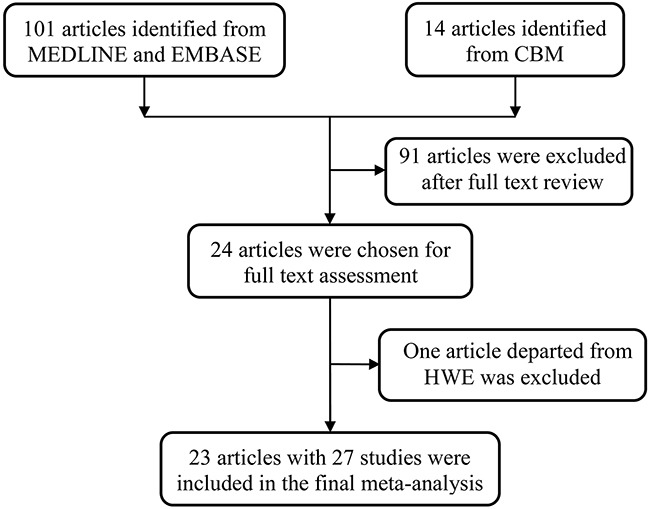
Flowchart of included studies

We have initially analyzed 115 potentially relevant publications. After full review, 91 articles were excluded for the following reasons: 1) they were review articles or meta-analyses, 2) they did not investigate association between the *FGFR4* rs351855 G>A polymorphism and cancer risk, 3) they were not case-control studies, or 4) had no enough data reported to calculate the odds ratios (ORs) and 95% confidence intervals (CIs). One article was further excluded because of the departure from Hardy–Weinberg equilibrium (HWE) [32]. Only 23 articles consisting of 27 individual studies investigated the association between the *FGFR4* rs351855 G>A polymorphism and cancer risk, and fit the eligibility criteria (Figure 1).

In total, data were obtained on 18,413 subjects (8,682 cases and 9,731 controls) from 27 studies (Table 1). The *FGFR4* rs351855 G>A polymorphism was most often studied in breast cancer (6 studies) [18–22] and prostate cancer (6 studies) [23–26]. Other commonly investigated tumor types were colorectal cancer (CRC, 3 studies) [18, 20, 33], hepatocellular carcinoma (HCC, 3 studies) [13, 30, 31], and lung cancer (2 studies) [28, 29]. Additionally, studies on head and neck squamous cell carcinoma (HNSCC) [27], oral squamous cell carcinoma (OSCC) [34], non-Hodgkin lymphoma (NHL) [35], gastric cancer [36], skin cancer [37], glioblastoma [38], and sarcoma [39] were combined and categorized as “others” cancer type, since only one study for each tumor type was reported. Fourteen studies utilized population-based (PB) controls and ten had hospital-based (HB) controls. Source of control was not determined (ND) in three studies. Studies were most frequently conducted among Caucasians (14 studies), followed by Asians (11 studies), and Africans (2 studies). Eleven studies were considered as low quality (quality score ≤ 9), and 16 studies (56%) were considered as high quality studies (quality score > 9).

**Table 1:** Characteristics of studies included in the current meta-analysis

Surname	Year	Cancertype	Country	Ethnicity	Design	Genotypemethod	Case				Control				MAF	HWE	Score
							GG	AG	AA	All	GG	AG	AA	All			
Bange	2002	Breast	Russia	Caucasian	PB	PCR-RFLP	26	28	7	61	55	60	8	123	0.31	0.114	7
Bange	2002	Breast	Germany	Caucasian	PB	PCR-RFLP	41	34	9	84	55	60	8	123	0.31	0.114	8
Bange	2002	CRC	Italy	Caucasian	PB	PCR-RFLP	37	38	7	82	55	60	8	123	0.31	0.114	8
Morimoto	2003	Sarcomas	Japan	Asian	NA	PCR-RFLP	54	72	17	143	39	50	13	102	0.37	0.624	6
Wang	2004	Prostate	USA	Caucasian	PB	PCR-RFLP	125	117	42	284	53	40	4	97	0.25	0.291	7
Wang	2004	Prostate	USA	African	PB	PCR-RFLP	37	6	2	45	76	18	0	94	0.10	0.305	6
Spinola	2005	Lung	Italy	Caucasian	HB	Pyrosequencing	148	103	23	274	193	168	40	401	0.31	0.699	11
Spinola	2005	Breast	Italy	Caucasian	HB	Pyrosequencing	67	55	20	142	112	83	25	220	0.30	0.117	10
Spinola	2005	CRC	Italy	Caucasian	HB	Pyrosequencing	98	63	18	179	112	83	25	220	0.30	0.117	10
Mawrin	2006	Glioma	Germany	Caucasian	HB	PCR-RFLP	39	51	4	94	10	13	2	25	0.34	0.428	10
Ma	2008	Prostate	Japan	Asian	HB	PCR-RFLP	163	196	133	492	67	87	25	179	0.38	0.701	10
Ansell	2009	HNSCC	Sweden	Caucasian	PB	PCR-RFLP	61		49^a^	110	81		111^a^	192	/	/	10
FitzGerald	2009	Prostate	USA	Caucasian	PB	SNPlex	587	544	123	1254	631	496	124	1251	0.30	0.070	15
FitzGerald	2009	Prostate	USA	African	PB	SNPlex	104	39	3	146	60	18	2	80	0.14	0.646	13
Ho	2009	HCC	Singapore	Asian	PB	Sequencing	27	17	14	58	30	38	20	88	0.44	0.241	8
Naidu	2009	Breast	Malaysia	Asian	HB	PCR-RFLP	179	172	36	387	132	105	15	252	0.27	0.322	9
Nan	2009	Skin	USA	Caucasian	PB	Taqman	365	325	78	768	406	343	84	833	0.31	0.359	12
Ho	2010	Prostate	UK	Caucasian	PB	Taqman	183	182	32	397	150	117	24	291	0.28	0.860	10
Tanuma	2010	OSCC	Japan	Asian	HB	PCR-SSCP	69	53	28	150	42	48	10	100	0.34	0.487	7
Batschauer	2011	Breast	Brazil	Caucasian	PB	PCR-RFLP	39	26	3	68	47	35	3	85	0.24	0.249	8
Heinzle	2012	CRC	Austria	Caucasian	PB	Taqman	190	148	25	363	802	723	135	1660	0.30	0.114	14
Yang	2012	HCC	China	Asian	HB	Taqman	216	351	144	711	247	361	132	740	0.42	0.996	13
Fang	2013	Lung	China	Asian	HB	Sequencing	193	331	105	629	163	391	175	729	0.51	0.049	11
Shen	2013	Gastric	China	Asian	PB	Sequencing	118	124	62	304	132	188	72	392	0.42	0.724	12
Gao	2014	NHL	China	Asian	NA	PCR-RFLP	117	189	115	421	171	240	75	486	0.40	0.541	8
Jiang	2015	Breast	China	Asian	NA	SNaPshot	205	404	138	747	270	348	98	716	0.38	0.398	11
Sheu	2015	HCC	China	Asian	HB	Taqman	82	150	57	289	159	314	122	595	0.47	0.146	10

Abbreviations: MAF, minor allele frequency; HWE, Hardy-Weinberg equilibrium, CRC, colorectal cancer; HNSCC, head and neck squamous cell carcinoma; HCC, hepatocellular carcinoma; OSCC, oral squamous cell carcinoma; NHL, non-Hodgkin lymphoma; PB, population based; HB, hospital based; PCR-RFLP, polymorphism chain reaction-restriction fragment length polymorphism; PCR-SSCP, polymorphism chain reaction-single-stranded conformation polymorphism.

^a^ data was provided for AG/AA.

### Association between *FGFR4* rs351855 G>A polymorphism and cancer risk

**Figure 2:**
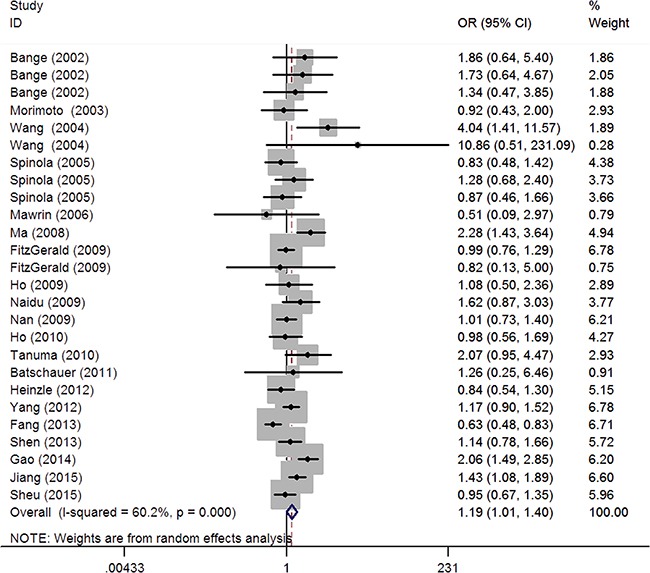
Forest plot of the association between the *FGFR4* rs351855 G>A polymorphism and cancer risk under the recessive model

The main findings of the meta-analysis are shown in Table 2 and Figure 2. Overall, the *FGFR4* rs351855 G>A polymorphism was associated with increased cancer risk under the recessive model (OR=1.19, 95% CI=1.01-1.41). The association appeared to be negative under the homozygous (OR=1.19, 95% CI=0.98-1.44), heterozygous (OR=1.00, 95% CI=0.91-1.10), dominant (1.02, 95% CI=0.91-1.13), and allele contrast models (OR=1.07, 95% CI=0.98-1.16). Our data suggest that individuals with AA genotype of the *FGFR4* rs351855 G>A polymorphism are at significantly increased cancer risk compared with AG and GG genotypes.

**Table 2:** Meta-analysis of the association between *FGFR4* rs351855 G>A polymorphism and cancer risk

Variables	No. of studies	Homozygous		Heterozygous		Recessive		Dominant		Allele	
		AA vs. GG		AG vs. GG		AA vs. AG/GG		AG/AA vs. GG		A vs. G	
		OR (95% CI)	*P*^het^	OR (95% CI)	*P*^het^	OR (95% CI)	*P*^het^	OR (95% CI)	*P*^het^	OR (95% CI)	*P*^het^
All ^a^	27	1.19 (0.98-1.44)	<0.001	1.00 (0.91-1.10)	0.012	**1.19 (1.01-1.41)**	<0.001	1.02 (0.91-1.13)	<0.001	1.07 (0.98-1.16)	<0.001
Cancer type											
Breast	6	**1.73 (1.35-2.20)**	0.960	1.17 (0.95-1.45)	0.186	**1.46 (1.17-1.83)**	0.986	**1.25 (1.02-1.52)**	0.197	**1.26 (1.14-1.41)**	0.622
CRC	3	0.84 (0.59-1.19)	0.701	0.87 (0.72-1.06)	0.964	0.89 (0.63-1.26)	0.718	0.87 (0.72-1.04)	0.895	0.90 (0.78-1.04)	0.773
Others	7	1.25 (0.85-1.84)	0.017	0.96 (0.81-1.14)	0.290	1.30 (0.92-1.85)	0.019	0.96 (0.77-1.18)	0.040	1.09 (0.92-1.30)	0.022
Prostate	6	1.60 (0.99-2.61)	0.020	**1.16 (1.02-1.32)**	0.714	1.56 (0.92-2.65)	0.004	**1.20 (1.06-1.35)**	0.892	**1.22 (1.06-1.41)**	0.183
Lung	2	**0.58 (0.40-0.83)**	0.231	**0.75 (0.61-0.91)**	0.596	**0.67 (0.53-0.85)**	0.388	**0.70 (0.58-0.85)**	0.332	**0.76 (0.67-0.86)**	0.358
HCC	3	1.08 (0.84-1.38)	0.341	0.93 (0.68-1.28)	0.127	1.09 (0.89-1.33)	0.656	0.96 (0.73-1.28)	0.139	1.02 (0.88-1.19)	0.241
Ethnicity											
Caucasians	14	1.05 (0.88-1.25)	0.349	1.04 (0.95-1.13)	0.497	1.02 (0.88-1.19)	0.448	1.00 (0.89-1.12)	0.116	1.03 (0.95-1.12)	0.192
Asians	11	1.25 (0.90-1.73)	<0.001	0.96 (0.80-1.16)	0.001	1.28 (0.99-1.66)	<0.001	1.03 (0.84-1.26)	<0.001	1.09 (0.93-1.28)	<0.001
African	2	2.17 (0.20-23.14)	0.169	1.05 (0.61-1.80)	0.322	2.21 (0.18-26.83)	0.019	1.11 (0.66-1.86)	0.617	1.15 (0.73-1.82)	0.948
Source of control											
PB	14	1.07 (0.90-1.27)	0.365	1.00 (0.88-1.13)	0.169	1.07 (0.92-1.25)	0.409	0.99 (0.87-1.12)	0.082	1.04 (0.96-1.12)	0.343
ND	3	**1.83 (1.30-2.56)**	0.175	**1.30 (1.03-1.64)**	0.218	**1.54 (1.08-2.22)**	0.088	**1.44 (1.19-1.76)**	0.283	1.34 (1.14-1.58)	0.170
HB	10	1.08 (0.77-1.51)	<0.001	0.92 (0.81-1.05)	0.237	1.13 (0.84-1.50)	<0.001	0.96 (0.81-1.13)	0.018	1.02 (0.87-1.18)	<0.001

Abbreviations: Het, heterogeneity; CRC, colorectal cancer; HCC, hepatocellular carcinoma; HB, Hospital based; PB, Population based; ND, not determined.

Analysis stratified by cancer type indicated that *FGFR4* rs351855 [A] carriers have modestly increased risk of developing breast cancer (homozygous: OR=1.73, 95% CI=1.35-2.20; recessive: OR=1.46; 95%CI=1.17-1.83; dominant: OR=1.25, 95% CI=1.02-1.52; allele contrast: OR=1.26; 95% CI=1.14-1.41), and prostate cancer (heterozygous: OR=1.16, 95% CI=1.02-1.32; dominant: OR=1.20, 95% CI=1.06-1.35; allele contrast: OR=1.22, 95% CI=1.06-1.41). Conversely, a modest reduction in cancer risk was found for lung cancer (homozygous: OR=0.58, 95% CI=0.40-0.83; heterozygous: OR=0.75, 95% CI=0.61-0.91, recessive: OR=0.67, 95% CI=0.53-0.85; dominant: OR=0.70, 95% CI=0.58-0.85; allele contrast: OR=0.76, 95% CI=0.67-0.86). When stratified analysis was performed by ethnicity, no association between ethnicity and cancer risk was observed among Caucasians, Asians, and Africans. Stratified analysis by source of control revealed significant association in ND subgroup (homozygous: OR=1.83, 95% CI=1.30-2.56); heterozygous: OR=1.30, 95% CI=1.03-1.64, recessive: OR=1.54, 95% CI=1.08-2.22; dominant: OR=1.44, 95% CI=1.19-1.76), rather than in PB and HB subgroups.

A significant heterogeneity existed in the overall pooled analysis for the association of interest under all genetic models, as indicated by the *P* values of heterogeneity test (*P*^het^) in the Table 2. Moreover, in the analyses stratified by cancer type, ethnicity, and source of control, significant heterogeneity was detected in prostate cancer, Asians, and in HB studies, respectively. Heterogeneity indicates diversity, which may result from differences in subjects, genotyping method, study design, sample size, ethnicity, and many other factors varying across studies. Therefore, the Mantel–Haenszel random effects model was used to decrease the effect of heterogeneity in these cases.

**Figure 3:**
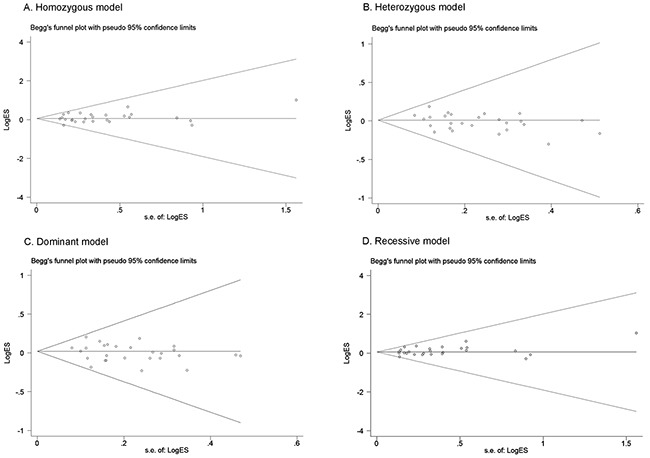
Funnel plots of *FGFR4* rs351855 G>A polymorphism Each point represents a separate study for the indicated association. **(A)** homozygous model; **(B)** heterozygous model; **(C)** dominant model; **(D)** recessive model.

We assessed validity of the results by sensitivity analyses and examination of potential publication bias. Sensitivity analysis revealed that no individual study substantially altered the pooled results, showing the stability of the present meta-analysis (Supplementary Figure 1). The meta-analysis was free of publication bias, as indicated by symmetrical funnel plots (Figure 3), and by the Egger's linear regression test (homozygous: *P*=0.461; heterozygous: *P*=0.085; recessive: *P*=0.229; dominant: *P*=0.27; allele contrast: *P*=0.938).

## DISCUSSION

The current meta-analysis demonstrates that the *FGFR* rs351855 G>A polymorphism is associated with an increased cancer risk. Consistent with our study, a previous meta-analysis in 2010, which included 12 studies with 4,892 cases and 3,663 controls, suggested that the *FGFR* rs351855 G>A polymorphism conferred increased genetic susceptibility to cancer [40]. The same meta-analysis also showed that the rs351855 G>A polymorphism significantly elevated the risk of prostate cancer and breast cancer [40]. However, in the analysis stratified by cancer type, only three studies for breast cancer were included, and no study for lung cancer was reported [40]. Since 2010, new studies have emerged. The current meta-analysis of 27 studies comprising 8,682 cases and 9,731 controls, confirmed the increased cancer risk of the rs351855 G>A polymorphism with increased statistical power. Moreover, our results indicate that the *FGFR4* rs351855 G>A polymorphism increases the risk of prostate and breast cancer, but decreases the risk of lung cancer. The reasons why the *FGFR4* rs351855 G>A polymorphism has opposite effects on different types of cancer are unclear, suggesting that other genetic and environmental factors might be involved, or that this polymorphism modifies cancer susceptibility in a tissue-specific manner. Alternatively, the association found with lung cancer might not be noteworthy, since only two studies were involved. Other two meta-analyses were performed regarding the *FGFR* rs351855 G>A polymorphism [41, 42]. However, one study focused on the association between the polymorphism and prognosis (nodal status and overall survival) [41], and the other analysis only included studies on prostate cancer [42].

Interestingly, the *FGFR* rs351855 G>A polymorphism genotypes are distributed differently among different ethnic groups [23, 40]. The current meta-analysis confirmed that the *FGFR* Arg^388^ allele is the most prevalent in Asians (40.1%), and then Caucasians (30.4%), and Africans (11.7%). However, analysis stratified by ethnicity failed to find any ethnic-specific association between the *FGFR* rs351855 G>A polymorphism and cancer risk. That is inconsistent with the previous meta-analysis study [40], which reported a significant association in Asians, with almost half the sample size (11 studies vs. 6 studies).

The biological effect of the *FGFR4* Arg388 expression remains unclear. However, several lines of evidence indicate the implication of the rs351855 G>A polymorphism in carcinogenesis. *In vitro* experiments with triple negative breast cancer cells (MDA-MB-231) demonstrated that cells expressing the *FGFR4* Arg388 variant have increased motility compared with cells expressing the *FGFR4* wide-type counterpart (Gly388) [18]. Wang et al. reported that compared to Gly388 cDNAs, transfection of *FGFR-4* Arg388 cDNAs promoted migration and invasion of PNT1A cells (prostatic epithelial cell line) [23].

However, no correlation has been established yet between the *FGFR4* rs351855 G>A genotype and FGFR4 protein levels. A previous study did not detect any correlation between the *FGFR4* rs351855 G>A genotype and FGFR4 protein expression among 104 HNSCC patients [43]. In addition, the Gly388Arg polymorphism did not alter FGFR4 protein expression in normal lung tissue [28]. FGFR4 serves as a receptor tyrosine kinase. However, the rs351855 G>A polymorphism did not alter the tyrosine kinase activity of FGFR4 in breast cancer [18] and prostate cancer cells [23]. Therefore, the SNP may increase cancer risk through other mechanisms, including altering FGFR4′s ligand affinity, degradation, or its capacity to interact with downstream effectors. In this regard, Tateno et al. found that the *FGFR4* polymorphism contributes to pituitary tumorigenesis through increasing phosphorylation of the mitochondrial STAT, resulting in increased cell growth [44]. Ulaganathan and Ullrich have recently reported that replacement of the glycine residue with a charged arginine residue at codon 388 causes changes in the transmembrane region, which consequently exposes a membrane-proximal cytoplasmic STAT3 binding site Y390-(P)XXQ393 [45]. Such *de novo* exposure of the STAT3 binding site facilitates STAT3 tyrosine phosphorylation, thereby stimulating cell proliferation [45]. Collectively, the *FGFR4* rs351855 G>A polymorphism may promote tumorigenesis by enhancing cell migration, invasion, and proliferation. More studies are needed to validate the association and investigate the underlying mechanisms. This SNP may hold a promise as a potential chemotherapeutic target and a biomarker to screen high-risk individuals.

There were some limitations in our meta-analysis study. First, the moderate sample size might have reduced the statistical power, especially in the stratified analyses. Second, because of the unavailability of demographic and environmental information, confounding factors could not be adjusted for, including age, sex, smoking, and drinking. Since the strength of the association was measured by crude ORs, our results might be open to confounding bias. Third, publication bias may be inevitable since we were only able to acquire data from unpublished articles. Finally, the meta-analysis was associated with a significant heterogeneity, which might weaken reliability of the meta-analysis.

In conclusion, our study demonstrates the association between the *FGFR4* rs351855 G>A polymorphism and overall cancer risk. In terms of cancer type, the *FGFR4* rs351855 G>A polymorphism was found to modify susceptibility to breast, prostate, and lung cancer.

## MATERIALS AND METHODS

The meta-analysis was performed according to the latest meta-analysis guidelines (PRISMA) [46].

### Data sources

We retrieved all the published studies relating the *FGFR4* rs351855 G>A polymorphism and cancer risk by searching PubMed and EMBASE databases. The combination of search terms were as follows: “FGFR4 or fibroblast growth factor receptor 4”, “rs351855 or Gly388Arg”, “polymorphism or variation or variant”, and “cancer or carcinoma or tumor”. The latest search was carried out in October 2016. We also examined the reference lists of the relevant original publications and review articles as well as previously published meta-analyses to maximize the coverage of the current meta-analysis. No language restriction was applied.

### Inclusion and exclusion criteria

Inclusion and exclusion criteria were predetermined as described previously [47–49]. To be included, studies had to: (i) be case-control or cohort studies, (ii) assess the association between the *FGFR4* rs351855 G>A polymorphism and cancer risk, and (iii) provide adequate data for calculating odd ratios (ORs) and 95% confidence intervals (CIs). We discarded case reports, case only studies, review articles, and conference abstracts. Additionally, we excluded studies that departed from Hardy–Weinberg equilibrium (HWE) (*P*_HWE_ < 0.05) in controls, if there was no extra evidence from another *FGFR4* polymorphism satisfying HWE. When duplicate studies occurred, only the most recent or the largest study was included.

### Data extraction

Two investigators (Fen Feng and Jianqun Ma) obtained data from individual studies independently following a standardized data extraction form. Data were extracted by name of the first author, year of publication, country where the study was conducted, ethnicity, genotype counts of cases and controls for the *FGFR4* rs351855 G>A polymorphism, source of controls (controls were chosen from the general population or a hospital), genotype method, and the *P*-value of HWE in controls. The subgroups were defined by cancer type, ethnicity, and source of control [hospital-based (HB) and population-based (PB)]. Publications containing different ethnicities, cancer types or different regions, were separated into different categories. The extracted information was imported into excel worksheets and examined for inconsistency. Conflicts were resolved by discussion between the two authors and a consensus would be reached ultimately.

### Quality assessment

The quality of studies was scored based on the following criteria: representativeness of case, representativeness of control, ascertainment of cancer case, control selection, genotyping examination, HWE, and total sample size as described in previous study [50]. The final quality score ranged from 0 (lowest) to 15 (highest). A score of 9 was used as a cutoff value (high quality: > 9; low quality: ≤ 9).

### Statistical analysis

A goodness-of-fit chi-square test was run to check departure from HWE for the *FGFR4* rs351855 G>A polymorphism in controls. The common measure of association across studies, the OR with 95% CI, was applied to the meta-analysis. The strength of the association between the *FGFR4* rs351855 G>A polymorphism and cancer risk was determined under the four genetic models (homozygous: AA vs. GG; heterozygous: AG vs. GG; recessive: AA vs. AG/GG; dominant: AG/AA vs. GG) and allele contrast was performed (A vs. G). We performed standard chi-square tests and the I^2^ statistic to quantify between-study heterogeneity [51]. If significance between-study heterogeneity was detected, summary ORs (95% Cls) were computed using a Mantel–Haenszel random effects model to decrease the effect of heterogeneity. If not, fixed effects model would take effect. Cancer type, ethnicity, and source of control were specified as study level characteristics for evaluating the source of heterogeneity using stratified analysis [52]. In order to explore the impact of each individual study on pooled risk estimates, we also carried out sensitivity analyses, which were fulfilled by leaving out one study at a time and recalculating ORs for the remaining studies. Publication bias was assessed through Begg's funnel plots [53] and Egger's linear regression test [54]. All analyses were conducted using STATA version 12 (Stata Corp, College Station, TX). All statistical analyses were two-sided, and a value of *P* <0.05 was considered significant.

## SUPPLEMENTARY FIGURE


