# Supplementary material for: Functional FGFR4 Gly388Arg polymorphism contributes to cancer susceptibility: Evidence from meta-analysis
Source: Oncotarget. 2017 Feb 28;8(15):25300–9. doi: 10.18632/oncotarget.15811 (PMC5421931; doi:10.18632/oncotarget.15811)
Supplement: Supplementary file 1 [file oncotarget-08-25300-s001.pdf]

Functional *FGFR4* Gly388Arg polymorphism contributes to cancer susceptibility: Evidence from meta-analysis

SUPPLEMENTRY FIGURE

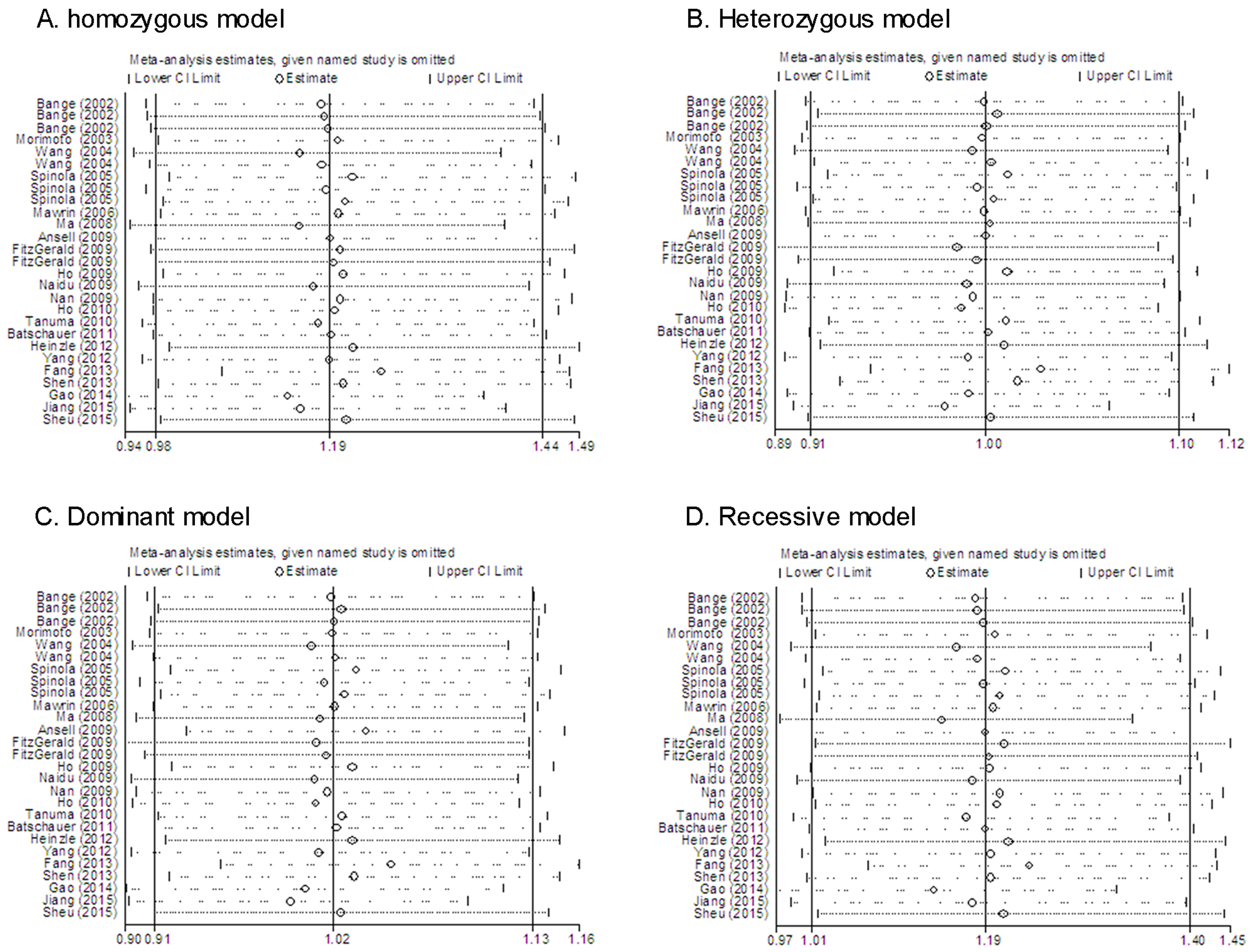

Supplementry Figure 1: Sensitivity analyses of potential publication bias of *FGFR4* rs351855 G>A polymorphism. (A) homozygous model; **(B)** heterozygous model; **(C)** dominant model; **(D)** recessive model.
